# Forms of Stigma and Discrimination in the Daily Lives of HIV-Positive Individuals in Mauritania

**DOI:** 10.2174/1874613601711010012

**Published:** 2017-03-31

**Authors:** Boushab Mohamed Boushab, Fatim-Zahra Fall-Malick, Mohamed Limame Ould Cheikh Melaïnine, Leonardo Kishi Basco

**Affiliations:** 1 Department of Internal Medicine and Infectious Diseases, Kiffa Regional Hospital, Assaba, Mauritania; 2 National Institute of Hepatitis and Virology, School of Medicine, Nouakchott, Mauritania; 3 Ambulatory Treatment Center, National Hospital Center of Nouakchott, Nouakchott, Mauritania; 4 Research Unit of Emerging Infectious and Tropical Diseases, Institut de Recherche pour le Développement, Aix-Marseille University, Marseille, France

**Keywords:** Stigma, Discrimination, AIDS, Mauritania, PLWHA, UNAIDS

## Abstract

**Summary:**

People living with HIV/AIDS (PLWHA) are often discriminated against in their daily lives. The objective of this descriptive and transversal study was to describe the experiences of PLWHA followed at a specialized outpatient center in Nouakchott to assess the forms of stigma from the perspective of those who suffer from discrimination.

**Methods:**

All HIV-positive patients over the age of 18 years who were aware of their HIV status and provided consent to participate in the study were included from June 1 to 29, 2015. Data collection was conducted using a pre-tested questionnaire.

**Results:**

A total of 210 PLWHA were interviewed. Men accounted for 54% of the sample population with a sex ratio of 1.2. About half of respondents were married (51%) and resided in Nouakchott (55%). Subjects who had never attended school represented 42% of the cases. Among our respondents, 64% knew their HIV status for over a year and admitted that they refused to reveal this information to any person. The distribution of forms of stigma experienced by PLWHA by demographic category was, in descending order, stigma in interpersonal relationships (78%), self-stigma (20%), and stigma in health services (2%). There was a significant association between the form of stigma and marital status (p = 0.007) and between the form of stigma and knowledge of HIV status for a period greater than one year (p = 0.02).

**Conclusion:**

The forms of stigma can be sources of discrimination and are a major obstacle to reintegration and support of PLWHA. This creates a vicious circle that, on the one hand, leads to the suffering, marginalization, and isolation of PLWHA, and on the other hand, has deleterious effects on their family and social relationships, self-esteem and self-confidence.

## INTRODUCTION

HIV/AIDS has become epidemic since the first cases were identified in the 1980s [1]. According to the 2014 UNAIDS report, 35 million people were living with HIV worldwide by the end of 2013, and the highest numbers of people living with HIV were in sub-Saharan Africa, with about 24.7 million people living with HIV/AIDS [2]. In Mauritania, the epidemiological situation of HIV infection is not well known. Nevertheless, existing data suggest an estimated seroprevalence rate of less than 1% in the general population [3]. Based on this prevalence rate, Mauritania can be considered as the least affected country in West Africa. PLWHA may be confronted with the belief that they have profoundly changed their lifestyles, and the comments received from them reflect a very serious concern about the deterioration of social ties. They are based on a representation of AIDS that produces an image of the PLWHA in Africa who are necessarily rejected and excluded by the entourage [4].

The sociological aspects of HIV/AIDS have not been studied on a large scale in Mauritania. The objective of the present study was to describe the experiences of PLWHA followed at a specialized outpatient treatment center in Nouakchott in order to evaluate the forms of stigma from the point of view of those who suffer from it.

## PATIENTS AND METHODS

The single-pass, descriptive cross-sectional study was carried out at the outpatient HIV-treatment center in Nouakchott during the period from June 1 to 29, 2015. This specialized care unit was designed to provide specific care and needs of patients infected with HIV. This care center was created with the support of the French Red Cross and is the only specific medical care structure in the country that offers PLWHA a range of complete care, including antiretroviral treatment. The study population consisted of HIV-positive patients aged more than 18 years old who were aware of their HIV status and provided a written informed consent to participate in the study. Data collection was performed using a previously tested questionnaire. The questions focused on socio-demographic attributes, clinical data, and data on the personal views of the PLWHA on his or her relationships with other family members. The diagnosis of HIV infection was established using the rapid diagnostic test DetermineTM HIV-1/2 (Alere Medical Co. Lt, Japan). Data were entered and analyzed using version 6.4 EPI INFO software. For the comparison of qualitative variables, the chi-square test was used. The P value of ≤ 0.05 was chosen as the threshold of significance.

## RESULTS

During our four-week study period, 210 PLWHA were interviewed. These patients were composed of 113 men (54%) and 97 women (46%), with a sex ratio (men:women) of 1.2. About half of the respondents were married (51%) and resided in Nouakchott (55%) at the time of inclusion. Among married patients, his or her partner was also HIV-positive in all cases, and they were living together at the time of inclusion. Subjects who had never attended school accounted for 42% of the cases. Among our interviewees, 64% knew their HIV status for more than a year and refused to reveal this information to any close friend or family members. The distribution of forms of stigma and discrimination perceived and experienced by PLWHA by socio-demographic category, in descending order, was as follows: stigma in interpersonal relationships (78%), self-stigma (20%), and stigma in health services (2%) (Table **1**). Despite the fact that 64% of patients refused to reveal their HIV status, the information was somehow revealed to a member of the entourage in additional 14% of the patients despite medical confidentiality. The relationships between the forms of stigma and socio-demographic categories are summarized in Table **2**.

## DISCUSSION

The HIV/AIDS epidemic has been the subject of several campaigns on information, education, communication and awareness in Mauritania, but stigma and discrimination still largely persist, and they increase the vulnerability of PLWHA [5]. The effects of stigma and discrimination have been addressed in psychiatry, social science, and public health on several occasions [6-10]. It is to the sociologist Erwin Goffman that we owe the first important study on this problem [11]. Subsequent studies have unanimously agreed that stigma has adverse effects on HIV/AIDS management [1, 12-14].

It is especially important to think of stigma as social and cultural phenomenon linked to the actions of whole groups of people in the developing world, where bonds and allegiances to families, village, neighborhood, and community abound [15, 16]. Rather than a static notion of stigma and discrimination, these phenomena should be analyzed and understood through new dynamic conceptual frameworks in different cultural backgrounds [15].

In our study, the sex ratio of male to female was 1.2. These results are likely to reflect the HIV/AIDS epidemic, which affects more men than women. This is mainly due to the steady increase in the proportion of men who have been infected through homosexual practices and poverty. Women living in poverty are less exposed to difficult conditions outside the home. More than half of the interviewees resided in Nouakchott, the capital city of Mauritania. This is due to a massive migration of the Mauritanian population to the capital where there is currently the only outpatient treatment center specifically created for medical care of the PLWHA in Mauritania. Furthermore, we found that the majority of HIV-infected individuals had never attended school or did not go beyond high school. This large percentage of uneducated or poorly educated patients may explain the lack of knowledge about the modes of transmission and HIV prevention and limited access to information on HIV, on the one hand, and vulnerability due to the risks that they unknowingly undertake in their sexual behavior, on the other hand [3, 5, 16]. Many of our interviewees (64%) had known their HIV status for more than a year but refused to reveal this information to intimate friends or family members. This refusal to communicate their medical condition can most probably be explained by the fear of being stigmatized by the society and the loss of esteem of the entourage [17-20]. Indeed, in much of the existing literature on stigma, investigators do not provide its definition at all, or refer to ‘a mark of disgrace’, or some similar aspect, such as stereotyping or social rejection. A consensus on the working definition of stigma and discrimination is required for further work on this problem [15]. Discrimination is a consequence of stigma and defined as “when, in the absence of objective justification, a distinction is made against a person that results in that person being treated unfairly and unjustly on the basis of belonging or being perceived to belong, to a particular group” [16]. In our study, stigma and discrimination in interpersonal relationships were the major form experienced by PLWHA. This form of stigma and discrimination seems to be more predominant in Mauritania than in other West African countries, such as Burkina Faso and Senegal, where studies have reported the prevalence of stigma in interpersonal relationships of 18% and 40%, respectively [18, 21]. The reason behind the importance of stigma and discrimination in interpersonal relationships observed in PLWHA in Mauritania may be due to the conception of the pathology itself by the population as a shameful disease since HIV is mainly transmitted by the sexual route in Africa. Neumann's analysis of stigma conducted in four African countries (Burkina Faso, Kenya, Malawi, and Uganda) showed that women were more likely to experience stigma in interpersonal relationships than men [22]. In our study, PLWHA who were separated or who had deceased partners were more likely to experience stigma in interpersonal relationships. The separation may have been a consequence of rejection by the partner, or widowhood may have been one of the causes of stigmatization. Our observations are similar to those reported in a study performed in Burkina Faso [18].

Self-stigmatization was less important in Mauritania, compared to other countries included in the MATCH study (Malawi, Ouganda and Kenya) and Burkina Faso [18, 22]. In other African countries, self-sigmatization was the most frequent form of stigma experienced by PLWHA. The detailed analysis of its manifestations showed that more than 20% of our PLWHA had sometimes low self-esteem due to HIV infection, indicating the limits of support for self-esteem among PLWHA [18]. Separated individuals and widows or widowers often lead stigmatized interpersonal relationships with or without HIV/AIDS, and are more likely to isolate themselves when infected and confronted with HIV/AIDS. This could be a consequence of or amplified by the stigma of which they are victims [23, 24].

Stigma in our health services was low. This result reflects a good level of acceptance of PLWHA by health workers. In our study, school-based PLWHA often reported that health care providers had taken more precautions with them than with other patients. These findings are similar to those reported in a study conducted in Burkina Faso [18].

We highlighted statistical differences related to marital status, gender, age, and sharing of confidentiality about the experience of stigma, suggesting that stigma affects all age groups, regardless of gender and the social environment. Nevertheless, as in other studies, the methodology does not make it possible to specify the meaning of this correlation. Associative community support and psychosocial care practices may place more emphasis on solidarity with patients than on the perception that PLWHA have of themselves in a country where there are very few psychologists in health care services.

## CONCLUSION

This study shows the difficulties of managing HIV/AIDS in Mauritania as in other African countries. From an applied point of view, these results show that a significant proportion of PLWHA suffer because of their status, despite the absence of stigmatization and discrimination by third parties, suggesting that the development of psychosocial counseling may allow more PLWHA to “live positively” with HIV. This requires financial resources for psychological support activities for HIV-infected persons, identification of innovative strategies for screening and treating people with depressive psychological disorders or high levels of self-stigma, which is a risk factor for clinical depression. Additional qualitative analyses are needed to define the role of associations, including the determination of whether associations' activities are ineffective in stigmatizing, or if participation in an association makes people more sensitive to discriminatory manifestations, which may explain why they are more effective, or if associations are a place of recourse for the people most affected by stigmatization. The strength of this study is its representativeness of PLWHA in Nouakchott because it has taken into account the PLWHA who met in different places of medical and community care but received care at the same clinic.

## FUNDING SOURCES

All authors declare that they have no funding sources.

## AUTHOR CONTRIBUTIONS

Boushab Mohamed Boushab (bboushab@gmail.com):1^st^ author and Corresponding author, he has been involved in drafting the manuscript, made substantial contributions to study conception and design, clinical data verification, discussion section.

Malick Fall Fatim-Zahra (zahrafallmalick@gmail.com): 2^nd^ author, manuscript correction.

Ould Cheikh Melaïnine Mohamed Limame (limameh@yahoo.fr): 3^th^ author, manuscript correction.

Basco Leonardo Kishi (lkbasco@yahoo.fr): 4^th^ author, manuscript correction.

All authors have read and approved the final manuscript.

## Figures and Tables

**Table 1:** Distribution of people living with HIV/AIDS by socio-demographic characteristics.

Socio-demographic Characteristics	Number	Percentage
Gender		
Male	113	54%
Female	97	46%
Marital status		
Married	107	51%
Divorced	55	26%
Single	39	19%
Widowed	9	4%
Age		
≥ 30 years old	178	85%
18 to < 30 years old	32	15%
Origin		
Nouakchott	116	55%
Other cities	94	45%
Education		
None	88	42%
Primary education	63	30%
Secondary education or higher	59	28%
Knowledge of privacy status and sharing		
Yes	134	64%
No	76	36%
Forms of stigma		
Interpersonal relationships	164	78%
Self-stigmatization	42	20%
Health care	4	2%

**Table 2:** Distribution of socio-demographic characteristics of people living with HIV/AIDS and forms of stigma.

Socio-demographic Characteristics	N	Self-stigmatization	Interpersonal Relationships	Health Care	P
Sexe					
Male	113	18	93	2	0.2
Female	97	24	71	2	
Marital status					
Married	107	34	71	2	0.07
Divorced	55	4	50	1	
Single	39	1	37	1	
Widowed	9	3	6		
Age					
≥ 30 years older	178	36	140	2	0.1
18 to < 30 years old	32	6	24	2	
Origin					
Nouakchott	116	24	90	2	0.9
Other cities	94	18	74	2	
Education					
none	88	15	70	3	
Primary education	63	15	48		
Secondary education or higher	59	12	46	1	
Knowledge of privacy status and sharing					
Yes	134	28	106		0.02
No	76	14	58	4	
